# Motor dysfunction in Parkinson’s patients: depression differences in a latent growth model

**DOI:** 10.3389/fnagi.2024.1393887

**Published:** 2024-06-03

**Authors:** QiuShuang Wang, Jing Bian, Yi Sun, YaoZhou Shi, ZiXuan Zhao, HuaShuo Zhao

**Affiliations:** ^1^Department of Biostatistics, School of Public Health, Xuzhou Medical University, Xuzhou, Jiangsu, China; ^2^The Affiliated Hospital of Xuzhou Medical University, Xuzhou, Jiangsu, China; ^3^Department of Orthopedics, First Clinical Medical College, Xuzhou Medical University, Xuzhou, Jiangsu, China; ^4^Department of Public Administration, School of Health Economics and Management, Nanjing University of Chinese Medicine, Nanjing, China

**Keywords:** Parkinson’s disease, latent growth model, motor dysfunction, depression, longitudinal study

## Abstract

**Objective:**

This study aims to utilize latent growth model (LGM) to explore the developmental trajectory of motor dysfunction in Parkinson’s disease (PD) patients and investigate the relationship between depression and motor dysfunction.

**Methods:**

Four-year follow-up data from 389 PD patients were collected through the Parkinson’s Progression Marker Initiative (PPMI). Firstly, a univariate LGM was employed to examine the developmental trajectory of motor dysfunction in PD patients. Subsequently, depression levels were introduced as covariates into the model, and depression was further treated as a parallel growth latent variable to study the longitudinal relationship between motor dysfunction and depression.

**Results:**

In the trajectory analysis of motor dysfunction, the fit indices for the quadratic growth LGM model were χ2 = 7.419, df = 6, CFI = 0.998, TLI = 0.997, SRMR = 0.019, and RMSEA = 0.025, indicating that the growth trend of motor dysfunction follows a quadratic curve rather than a simple linear pattern. Introducing depression symptoms as time-varying covariates to explore their effect on motor dysfunction revealed significant positive correlations (β = 0.383, *p* = 0.026; β = 0.675, *p* < 0.001; β = 0.385, *p* = 0.019; β = 0.415, *p* = 0.014; β = 0.614, *p* = 0.003), suggesting that as depression levels increase, motor dysfunction scores also increase. Treating depression as a parallel developmental process in the LGM, the regression coefficients for depression intercept on motor dysfunction intercept, depression slope on motor dysfunction slope, and depression quadratic factor on motor dysfunction quadratic factor were 0.448 (*p* = 0.046), 1.316 (*p* = 0.003), and 1.496 (*p* = 0.038), respectively. These significant regression coefficients indicate a complex relationship between depression and motor dysfunction, involving not only initial level associations but also growth trends over time and possible quadratic effects.

**Conclusion:**

This study indicates a quadratic growth trajectory for motor dysfunction in PD, suggesting a continuous increase in severity with a gradual deceleration in growth rate. The relationship between depression and motor dysfunction is complex, involving initial associations, evolving trends over time, and potential quadratic effects. Exacerbation of depressive symptoms may coincide with motor function deterioration.

## 1 Introduction

Parkinson’s disease (PD) is a chronic progressive neurological disorder, with its prevalence increasing with age. The World Health Organization predicts that by 2030, the global number of PD patients will reach approximately 8.67 million, with around 4.94 million in China (Ma et al., 2014). By 2040, the diagnosis numbers are expected to double (Titova and Chaudhuri, 2018). The main pathological changes in PD involve degeneration and reduction of dopaminergic neurons in the substantia nigra pars compacta (Munhoz et al., 2015). Dopamine, a neurotransmitter, is crucial for coordinating and controlling motor function. Therefore, the decline in dopamine levels directly affects motor function. The primary clinical symptoms of PD are motor disturbances, including bradykinesia, resting tremor, rigidity, postural instability, and dystonia. Bradykinesia is the most typical clinical feature of PD, usually manifested as a slowing of voluntary movements, reduced speed and amplitude of repetitive movements, drooling, masked face, and micrographia. Motor symptoms typically begin on one side of the body and then progress to the other side over several years (Postuma et al., 2015a; Xu et al., 2019). These symptoms significantly impact patients’ everyday mobility. Alongside the direct motor symptoms, PD is often accompanied by a range of non-motor symptoms (Zesiewicz, 2019), including cognitive impairment, mild autonomic dysfunction, depression, and anxiety. Some non-motor symptoms and signs have been shown to manifest several years or even decades before a clinical diagnosis of PD (Postuma et al., 2015b; Heinzel et al., 2019). These non-motor symptoms may indirectly affect patients’ motor function by influencing their overall quality of life and activity levels, extending well beyond the impairment of motor function (Lees et al., 2009; de la Riva et al., 2014; LeWitt and Chaudhuri, 2020). Non-motor symptoms persist throughout the course of PD and often manifest as initial clinical presentations (Weintraub et al., 2022). They do not necessarily disappear after the onset of motor symptoms; in fact, they may gradually worsen as the disease progresses. Among these non-motor symptoms, depression is particularly prevalent in PD patients, occurring in approximately 30% of cases (Jellinger, 1999) The onset of depressive symptoms can vary widely, ranging from as little as 1 month to as long as 30 years (Iranzo et al., 2014; Walter et al., 2015). Depression may precede the onset of Parkinson’s motor impairments, but in the subsequent course of development, depression and motor dysfunction may mutually influence each other. Currently, research on motor dysfunction in PD primarily focuses on mechanisms, functional assessment, monitoring, and treatment, with relatively few studies investigating the trajectory of motor function in Parkinson’s patients. Currently, numerous studies have investigated the relationship between motor and non-motor functions, including depression (Zahodne et al., 2012), autonomic dysfunction (Qin et al., 2024), cognitive impairment (Moustafa et al., 2016), olfactory dysfunction (Nabizadeh et al., 2022), and sleep disturbances (Bugalho and Viana-Baptista, 2013), all of which have demonstrated close associations with motor function. Among these factors, sleep disturbances are particularly intertwined with motor function, and a bidirectional relationship exists between sleep disturbances and depression, underscoring the importance of further exploring the relationship between depression and motor function (Fang et al., 2019).

Research on the motor function trajectory of PD patients and the relationship between depression and motor trajectory is relatively scarce. Cubo et al. (2000) employed univariate logistic regression analysis to examine the association between motor dysfunction assessed by total the Unified Parkinson’s Disease Rating Scale scores and Hoehn-Yahr staging and depression. Darweesh et al. (2017) conducted a nested case-control study, gathering data on daily functional trajectories and motor and non-motor features from 1990 to 2013, spanning 23 years before PD diagnosis. They utilized extended mixed models and found that PD patients increasingly displayed low and slow motor signs throughout the study follow-up compared to controls from 7.5 years before diagnosis, with statistically significant overall differences. However, this study has limitations as motor features were assessed by research nurses subjectively, using subjective ratings rather than specialized quantitative methods. Poonja et al. (2021), aiming to examine and describe the trajectory of Unified Parkinson’s Disease Rating Scale motor scores (UPDRS-III) in the late stages of PD, conducted separate modeling of UPDRS-III scores using piecewise linear models and clustered the resulting trajectories based on their characteristics. They concluded that while different populations with varying motor function in PD exhibited different changes, such as continuous deterioration, stable-deterioration, improvement-deterioration, and deterioration-improvement-deterioration, the eventual decline in motor function was universal. This study was a retrospective chart review rather than a data modeling study. Zahodne et al. (2012) obtained data from 186 PD patients (mean duration of disease 8.2 years) from a clinical research database, incorporating 18 months of data to examine the trajectories of motor symptoms and depressive symptoms. Unconditional univariate growth models indicated that over the 18-month observation period, both motor symptoms and apathy exhibited linear deterioration, while depressive symptoms initially showed improvement before worsening, demonstrating quadratic change. The study had a relatively short longitudinal follow-up period, a small sample size, and employed a rather simplistic model. However, the latent growth model (LGM) used in this study typically refers to a statistical approach that allows for consideration of individual differences (Curran et al., 2010), providing a better understanding of the patterns of development trajectories and changes among different individuals, effectively capturing the collective trajectories of individuals’ changes over time, suitable for long-term data analysis, and facilitating the understanding of dynamic changes in variables over time. LGM allows for the estimation of latent variables, revealing the underlying structure and relationships behind observed variables, thus providing a more comprehensive understanding of the data. It includes repeated measurements within individuals or multilevel data, making it more suitable for complex data analysis. This study selected 4-year longitudinal survey data of Parkinson’s disease patients from the Parkinson’s Progression Markers Initiative (PPMI)database, analyzing the changes in early motor function of PD patients using LGM. Furthermore, the study investigated the predictive level of depression on the developmental trajectory of motor function. Analyzing motor function trajectories can identify trends of motor function degradation in patients, enabling early intervention. Additionally, the association between depression and motor function degradation identified in the study also provides clues for early intervention. Early identification and treatment of depressive symptoms may help delay the progression of motor function degradation, contributing to the development of more comprehensive treatment plans and improving patients’ quality of life.

## 2 Subjects and methods

### 2.1 Subjects

**FIGURE 1:**
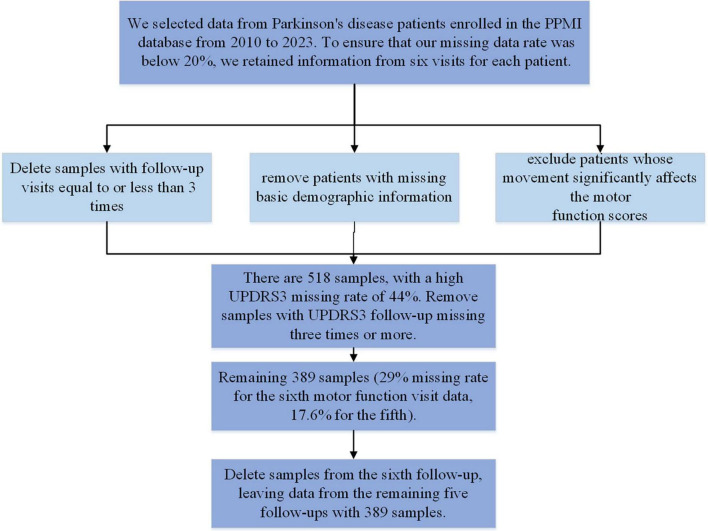
Sample selection.

We obtained data from the Parkinson’s Progression Markers Initiative (PPMI), a publicly available database. In 2010, The Michael J. Fox Foundation and a core group of academic scientists and industry partners launched the PPMI aims to investigate much-needed biomarkers for the onset and progression of PD. Data used in the preparation of this article were obtained from the PPMI database,^1^ RRID:SCR_006431. For up-to-date information on the study, visit www.ppmi-info.org. We included PPMI data from 2010 to 2023, collected at 12-month intervals. We included patients diagnosed with PD, excluding those with ≤3 follow-up visits and those with missing basic demographic information. We also removed data where motor function significantly affected motor function assessment. The sample selection process is outlined in Figure 1. After screening, a total of 389 samples remained. The maximum missing rate for depression data across the five follow-ups is 7.5%, and for motor function, it is 17.6%. Both sample size and missing data rates meet the modeling requirements. None of our participants received treatment at baseline, but underwent confirmative assessments, including clinical and cognitive evaluations, imaging examinations, and biological sampling, which were approved by the local participant Central Institutional Review Board. All participants provided written informed consent prior to enrollment. The sample included in this study and the code can be found in the Supplementary material.

### 2.2 Measurements

Depression levels were assessed using a condensed version of the Geriatric Depression Scale (GDS), namely the GDS-15. This scale comprises 15 items designed to gauge symptoms of depression, diminished activity, irritability, withdrawal from social engagements, as well as negative ruminations concerning the past, present, and future. Scores on the GDS-15 range from 0 to 15. Scores of 0–4 indicate normalcy; 5–8 suggest mild depression; 9–11 indicate moderate depression; and 12–15 signify severe depression. A score of 5 or higher suggests the likelihood of depression. The GDS-15 exhibits robust reliability and validity when administered to older adults (de Craen et al., 2003).

The Movement Disorder Society-Unified Parkinson’s Disease Rating Scale Part III (MDS-UPDRS3) is a scale used to assess motor dysfunction in PD and is one of the standard tools for clinical evaluation of the condition. MDS-UPDRS3 comprises a series of items covering four main aspects of motor function: postural control, bradykinesia, upper limb and trunk function, and gait and balance. These items are graded on a scale from 0 to 4, where 0 represents no symptoms and 4 represents severe impairment. The minimum score is 0, and the maximum score is 132. The total score reflects the severity of motor dysfunction in patients, with higher scores indicating more severe impairment. Widely utilized in both clinical practice and research, MDS-UPDRS3 is considered a reliable and effective tool for assessing motor function in PD patients (Weintraub and Mamikonyan, 2019).

### 2.3 Statistical analysis

Descriptive statistics were performed on the data, with categorical variables analyzed using frequency counts and continuous variables presented as means ± standard deviations. Pearson correlation analysis was conducted to explore the correlation coefficients between variables. The trend of motor dysfunction was observed, and then an appropriate LGM was selected for fitting, based on fit indices. In constructing the LGM, an unconditional model was first established, forming repeated measurements of motor trajectories across five time points. The linear growth model included two latent variables: intercept and slope. The mean and variance parameters of these latent variables were used by the LGM to describe within-group and between-group differences. Specifically, the mean of the intercept factor represented the average initial state, while the variance of the intercept factor indicated the extent of individual differences at specific time points. A greater variance suggests more significant initial differences between individuals. The mean of the slope factor represented the average growth rate between time points, and the variance of the slope factor reflected the magnitude of individual differences in growth rates. The bidirectional arrows between the two factors indicated their correlation. Next, a quadratic growth model was constructed, which added a Quadratic Slope to the linear growth model, representing the quadratic trend of the latent variable, i.e., the variable’s quadratic change over time. The quadratic factor described the concave or convex shape of the curve. These factors combined to model the trajectory of latent variables’ changes over time. This study examines the trajectory of motor dysfunction change, including significant individual differences in starting level, change trends, and quadratic changes. Additionally, in the model, depression (time-varying covariate) is included to explore the predictive effect of different levels of depression on the trajectory of motor dysfunction change in PD patients.

For the missing data, we use Maximum likelihood (ML) estimation to estimate the parameters of the model. To assess the model fit degree, we rely on Comparative Fit Index (CFI), Tucker-Lewis Index(TLI), Root Mean Square Error of Approximation(RMSEA) and Standardized Root Mean Square Residual(SRMR) values, as the χ2 goodness-of-fit statistic can be overly sensitive for large sample sizes, and therefore, we do not employ it. For CFI and TLI, a value above 0.90 is considered acceptable, and a value exceeding 0.95 indicates a good fit. RMSEA value below 0.05 indicates a good fit to the data, while a value between 0.05 and 0.08 suggests a suitable fit (Hu and Bentler, 1999). SRMR examines the fit of the model by the size of the residue, and its values range from 0 to 1 and indicate a good model fit when the value is less than 0.08.

To implement the necessary model and conduct the analysis, we utilize Mplus 8.9. Descriptive analysis and plotting are performed using SPSS version 25.0 and R (version 4.2.3). The test level is set at a p-value of 0.05.

## 3 Results

### 3.1 Preliminary analysis

It can be observed that in our sample (Table 1), there were 244 males (62.7%) and 145 females (37.3%). The onset age was predominantly above 56 years old (71.2%), with the majority being Caucasian (92.3%). Most individuals have had 13–23 years of education (81.3%). The average onset age was 59.08 ± 9.70 years old, and the duration of illness was 1.11 ± 1.44 years.

**TABLE 1:** Demographic information of the study participants (*n* = 389).

Variables	*N*	%
**Age**		
<56 years old	112	28.8%
56∼65 years old	125	32.1%
>65 years old	152	39.1%
**Gender**		
Female	145	37.3%
Male	244	62.7%
**Years of education**		
<13 years	67	17.2%
13–23 years	316	81.3%
>23 years	6	1.5%
**Race**		
White	359	92.3%
No white	30	7.7%
**Family history**		
Yes	129	33.2%
No	260	66.8%
Age at PD symptom onset	59.08 ± 9.70	
Duration from PD diagnosis	1.11 ± 1.44	

Age at PD symptom onset and duration from PD diagnosis are continuous variables. We describe them using mean ± standard deviation.

In Table 2, we can observe that the means and standard deviations of both variables are gradually increasing, indicating a progressive worsening of both motor dysfunction and depression levels as the disease progresses. Additionally, at each time point, there is a significant positive correlation between depression levels and motor dysfunction, with correlations being evident (*r* = 0.104, *p* < 0.05; *r* = 0.167, *p* < 0.01; *r* = 0.161, *p* < 0.01; *r* = 0.136, *p* < 0.05).

**TABLE 2:** The average level and bivariate correlations of the main study variables.

Variables (*N* = 389)	M ± SD										
updrs3BL	21.01 ± 9.243	1									
updrs3V04	24.99 ± 11.076	0.688**	1								
updrs3V06	27.35 ± 11.464	0.572**	0.688**	1							
updrs3V08	29.13 ± 12.556	0.507**	0.639**	0.705**	1						
updrs3V10	30.84 ± 12.933	0.457**	0.591**	0.630**	0.765**	1					
gdsBL	2.47 ± 2.640	0.104*	0.095	0.023	0.042	0.058	1				
gdsV04	2.57 ± 2.775	0.077	0.167**	0.076	0.047	0.106	0.700**	1			
gdsV06	2.80 ± 2.850	0.040	0.080	0.080	0.089	0.070	0.643**	0.674**	1		
gdsV08	2.88 ± 3.066	0.052	0.104	0.100	0.161**	0.171**	0.558**	0.630**	0.717**	1	
gdsV10	2.91 ± 3.070	0.074	0.098	0.049	0.112*	0.136*	0.504**	0.581**	0.686**	0.717**	1
		updrs3BL	updrs3V04	updrs3V06	updrs3V08	updrs3V10	gdsBL	gdsV04	gdsV06	gdsV08	gdsV10

**Significant at the 0.01 level (two-tailed).

*Significant at the 0.05 level (two-tailed).

### 3.2 Trajectories of motor dysfunction in Parkinson’s patients

Based on the changes observed in the basic statistical descriptions of depression and motor function scores, we proposed two trends: one linear and the other quadratic. The fit indices for the LGM were χ2 = 35.257, df = 10, CFI = 0.966, TLI = 0.966, SRMR = 0.046, and RMSEA = 0.081. For the quadratic LGM, the fit indices were χ2 = 7.419, df = 6, CFI = 0.998, TLI = 0.997, SRMR = 0.019, and RMSEA = 0.025. Comparing the linear LGM and quadratic LGM models, we found that the quadratic LGM model outperformed the linear model, indicating that the quadratic LGM model better fits the trajectory of our data’s development.

**FIGURE 2:**
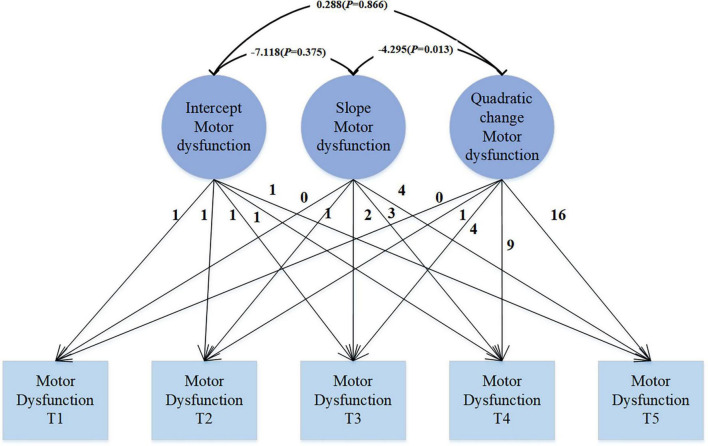
Unconditional quadratic LGM.

The quadratic growth LGM model (see Figure 2) reveals that at the starting point of observation, namely the initial state, the average intercept of PD motor function is 21.061 (SE = 0.477, *P* < 0.001), which significantly differs from 0, indicating an average score of 21.061 for motor dysfunction. The variance is 75.419 (SE = 9.845, *P* < 0.001), suggesting significant individual differences at the initial state, indicating considerable variability among individuals in the initial level of motor dysfunction. Over the course of 4 years, the mean slope of motor function is 3.733 (SE = 0.401, *P* < 0.001), reflecting a linear growth trend in motor dysfunction throughout the observation period. The variance is 25.759 (SE = 8.106, *P* = 0.001), indicating significant variability among individuals, even though the mean suggests an overall increasing trend, there are significant differences in the growth rates among individuals. The mean of the quadratic slope is −0.327 (SE = 0.099, *P* = 0.001), indicating that the growth trend of motor dysfunction is not a simple linear increase, but rather follows a quadratic curve. The variance is 0.965 (SE = 0.420, *P* = 0.021), indicating significant variability in the quadratic growth trend among individuals. The significant negative quadratic change implies that the increase in the severity of motor dysfunction gradually slows down over time. There is a significant correlation between the slope and quadratic change (*r* = −4.295, *p* = 0.013), with the negative correlation indicating a decreasing trend in quadratic change as the slope increases. The correlation between intercept and slope, as well as intercept and quadratic change (*r* = −7.118, *p* = 0.375; *r* = 0.288, *p* = 0.866), is not significant.

### 3.3 Time-specific effect of depression status on motor dysfunction in Parkinson’s patients

**FIGURE 3:**
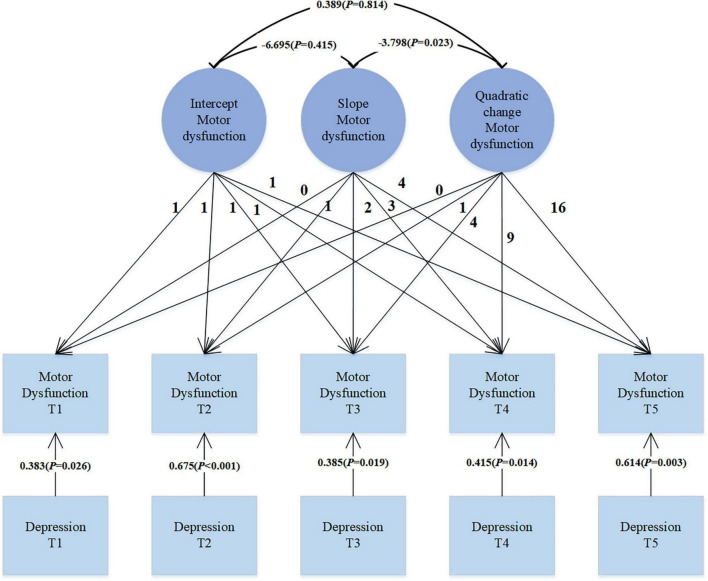
Quadratic LGM with time-varying covariates.

In our analysis, we introduced depression symptoms as a time-varying covariate to explore its impact on motor dysfunction. As shown in Figure 3, the data fit well: χ2 = 16.525, df = 26, CFI = 1.000, TLI = 1.017, SRMR = 0.029, RMSEA < 0.001. Depression is significantly correlated with motor dysfunction (β = 0.383, *p* = 0.026; β = 0.675, *p* < 0.001; β = 0.385, *p* = 0.019; β = 0.415, *p* = 0.014; β = 0.614, *p* = 0.003). This positive correlation indicates that as the level of depression increases, motor dysfunction scores also increase correspondingly. This may suggest that exacerbation of depression symptoms in PD patients may be accompanied by deterioration in motor function.

### 3.4 Trajectories of depression and motor dysfunction in Parkinson’s patients

**FIGURE 4:**
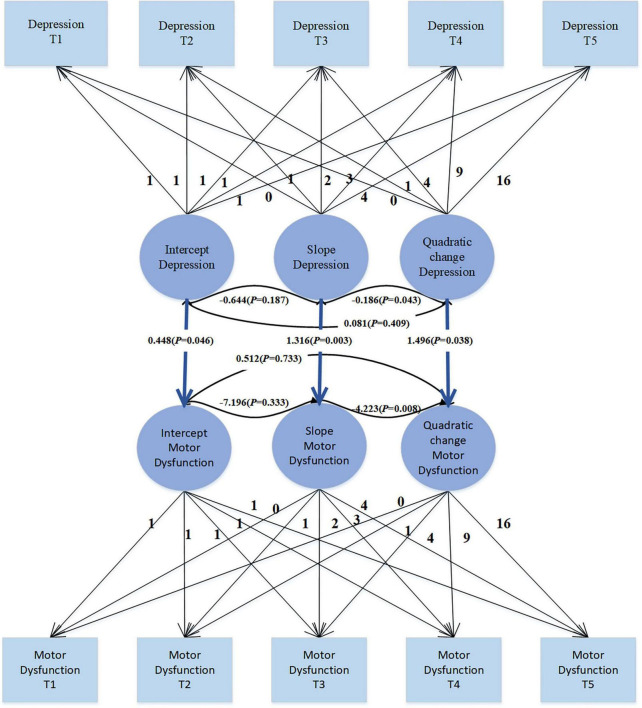
Parallel LGM of depression and motor dysfunction.

The use of covariates in parallel developmental processes’ LGM to study their impact on PD patients’ motor dysfunction, as shown in Figure 4, enables a comprehensive consideration of the developmental trajectories of covariates. This method not only allows for understanding the effects of covariates on the target variable but also facilitates understanding the trends in covariates themselves. Moreover, this approach is flexible in capturing the complex relationships between covariates and the target variable, including nonlinear and asymmetric relationships. The unconditional LGM with multiple processes fits the data well: χ2 = 29.876, df = 30, CFI = 1.000, TLI = 1.000, SRMR = 0.025, RMSEA < 0.001. In the model, the regression coefficient of depression intercept on motor dysfunction intercept is 0.448 (SE = 0.225; *P* = 0.046), indicating that individuals with higher levels of depression at the initial moment (intercept) also exhibit higher levels of motor dysfunction. The regression coefficient of depression slope on motor dysfunction slope is 1.316 (SE = 0.437; *P* = 0.003), suggesting an overall positive correlation between the growth trends of depression and motor dysfunction. This implies that individuals with increasing levels of depression over time are more likely to experience an increase in motor dysfunction levels. The regression coefficient of depression quadratic slope on motor dysfunction quadratic slope is 1.496 (SE = 0.721; *P* = 0.038), indicating the consideration of depression’s quadratic effect in the model, suggesting a positive correlation between the rates of change in depression’s growth trend and motor dysfunction’s growth trend. Overall, these significant regression coefficients suggest a complex relationship between depression and motor dysfunction, including not only the association at the initial level but also the growth trends over time and potential quadratic effects. This provides some clues for understanding the dynamic relationship between depression and motor dysfunction.

## 4 Discussion

First, this study employed a univariate LGM to explore the trajectory of motor dysfunction in PD patients. From the findings, our trajectory appears to follow a quadratic curve. Previous studies have indicated a linear trajectory for motor function (Zahodne et al., 2012; Poonja et al., 2021), but these studies only had data for 18 months. In contrast, we have data spanning 4 years, and from this, it seems that the severity of motor dysfunction gradually increases, albeit at a slowing rate. This may suggest that the progression of the disease slows down or stabilizes at a certain stage. May be due to the motor and depression scales we utilized have potential ceiling effects. The quadratic trend might be partly attributed to the scaling properties of the measurement tools, where scores may plateau, reflecting an upper limit of the scale rather than a true stabilization of symptoms. Nonetheless, the deterioration of motor function is inevitable. Therefore, closely monitoring changes in symptoms, adjusting treatment plans promptly, and taking measures to improve patients’ quality of life remain essential. Subsequently, we introduced depression as a covariate into our model, and further incorporated depression as a parallel developing LGM. This approach allows for a comprehensive consideration of the developmental trajectories of covariates, enabling us to understand not only the impact of covariates on the target variables but also the trends in the covariates themselves. Moreover, this method offers greater flexibility in capturing the complex relationships between covariates and target variables. Both models indicate that depression influences the trajectory of motor dysfunction to some extent. Whether depression is considered as a covariate changing over time or as a parallel developing LGM, it demonstrates an impact on motor function. The positive significance of the results suggests that depression may be a contributing factor to or exacerbate motor dysfunction to a certain extent. Depression is not merely a short-term effect but may have a sustained impact on motor function throughout the course of the disease. Therefore, in the treatment and management of PD patients, it is essential to consider the influence of depression comprehensively and take measures promptly to manage depressive symptoms in order to improve both motor function and quality of life for patients.

The mechanism underlying motor dysfunction in PD is primarily attributed to biochemical abnormalities in the basal ganglia and disruption of circuit activity. Degeneration of the nigrostriatal dopamine pathway in the substantia nigra to striatum leads to excessive output from the basal ganglia, while excessive inhibition of thalamocortical feedback activity impairs the facilitatory action of cortical motor function, resulting in motor disturbances. The basal ganglia exhibit intricate fiber connections, primarily comprising three crucial neural circuits: the corticocortical circuit involving the cerebral cortex, caudate nucleus, globus pallidus, thalamus, and cerebral cortex; the nigrostriatal circuit connecting the substantia nigra with the striatum and vice versa; and the striatopallidal circuit involving the caudate nucleus, putamen, external segment of the globus pallidus, subthalamic nucleus, and internal segment of the globus pallidus. These circuits form the anatomical basis for the motor regulation function of the basal ganglia, with the balanced activity of these pathways being vital for normal motor function. Treatment of motor disorders in PD, whether pharmacological or surgical, is based on correcting neurotransmitter abnormalities and disrupting circuit activity. Mechanistic studies of depressive symptoms also involve the striatum (Mulders et al., 2022), prefrontal cortical regions (especially the dorsolateral prefrontal cortex), as well as bottom-up subcortical networks (including the amygdala) (Almeida et al., 2009; He et al., 2019), and the hypothalamic-pituitary-adrenal axis (Liu et al., 2021). Additionally, neurodegeneration is widespread in brainstem nuclei, including autonomic areas such as the dorsal motor nucleus of the vagus nerve, motor nuclei like the pontine nuclei, and regulatory nuclei such as the locus coeruleus and raphe nuclei (Seidel et al., 2015). This correlates with both motor dysfunction and depressive symptoms. Therefore, there are some overlaps between the mechanisms of the two conditions, suggesting that depression may exacerbate or worsen motor dysfunction by affecting the neurotransmitter systems and neuronal activity in the brain, thereby influencing the neurotransmitter systems involved in motor control. Our study provides evidence for the association between the two, but further research is needed to elucidate the specific mechanisms between them. Currently, the main treatments for depression include the use of antidepressant medications, electroconvulsive therapy (Xu et al., 2020), repetitive transcranial magnetic stimulation (Dubovsky et al., 2021), etc. Additionally, exercise therapy (Cheng et al., 2016; LaHue et al., 2016) is a non-pharmacological treatment for depression, offering advantages such as affordability, convenience, high compliance, and minimal adverse reactions. Exercise therapy not only improves the worsening of depressive symptoms but also enhances motor function (Kashif et al., 2022). With the continuous advancement of medical technology, more comprehensive and detailed treatment strategies have been proposed, such as comprehensive rehabilitation for elderly patients with PD (Hongxia, 2023). This comprehensive rehabilitation model provides tailored rehabilitation assessments and treatment plans for elderly PD patients at different stages, aiming to comprehensively and scientifically manage the rehabilitation needs of patients. Covering the entire disease process of elderly PD patients, this comprehensive rehabilitation model can effectively improve physical function, mental health, and quality of life, delay disease progression, enhance coping abilities, and is an essential component of integrated treatment.

This study also has some limitations. Firstly, our study participants were mainly Caucasian, which may limit the generalizability of our findings. Secondly, our data were based on a 4-year period for LGM analysis. Although this provides valuable insights, there may be limitations for those interested in longer-term trajectory analyses. Future research could consider continuing follow-ups and increasing the frequency of assessments to obtain more comprehensive and reliable results. Thirdly, regarding the measurement of motor dysfunction, while the scales we used can accurately measure, there are updated techniques available for more precise long-term monitoring of motor symptoms in PD patients throughout the day or over several days to assess the range and severity of motor dysfunction symptoms. Therefore, if feasible, we could adopt more optimized methods in the future. Additionally, classifying PD patients according to different subtypes could provide more detailed insights into the trajectory of motor dysfunction for each subtype, warranting further investigation. Furthermore, exploring the relationship and mutual influence between motor dysfunction and depression in PD patients using other models is also worth considering.

## 5 Conclusion

Through the analysis of 4-year longitudinal data from PPMI, the developmental trajectory of motor dysfunction in PD patients was elucidated. Furthermore, the relationship between depression severity and motor dysfunction was revealed using a bivariate latent growth curve model. The findings indicate that the growth trajectory of motor dysfunction follows a quadratic curve rather than a simple linear increase. Specifically, while the severity of motor dysfunction continues to rise, the rate of increase gradually slows down. In the model of depression and motor dysfunction, all regression coefficients were found to be significant and positively correlated, suggesting a complex relationship between depression and motor dysfunction. This relationship encompasses not only the initial level of association but also the growth trend and possible quadratic effects over time. The exacerbation of depressive symptoms may be accompanied by worsening motor function, providing valuable insights into understanding the dynamic relationship between depression and motor dysfunction. Given their interaction, interventions aimed at improving depression symptoms in PD patients may contribute to the rehabilitation of motor dysfunction.

## Data availability statement

The original contributions presented in this study are included in this article/Supplementary material, further inquiries can be directed to the corresponding author.

## Author contributions

QW: Conceptualization, Data curation, Investigation, Software, Writing – original draft. JB: Formal analysis, Project administration, Writing – original draft. YiS: Data curation, Software, Writing – original draft. YaS: Investigation, Methodology, Writing – original draft. ZZ: Writing – review and editing. HZ: Writing – review and editing.

## Footnotes

1
www.ppmi-info.org/access-data-specimens/download-data

## References

[B1] Almeida J. R. Akkal D. Hassel S. Travis M. J. Banihashemi L. Kerr N. (2009). Reduced gray matter volume in ventral prefrontal cortex but not amygdala in bipolar disorder: Significant effects of gender and trait anxiety. *Psychiatry Res.* 171 54–68. 10.1016/j.pscychresns.2008.02.001 19101126 PMC2646161

[B2] Bugalho P. Viana-Baptista M. (2013). REM sleep behavior disorder and motor dysfunction in Parkinson’s disease–a longitudinal study. *Parkinsonism Relat. Disord.* 19 1084–1087. 10.1016/j.parkreldis.2013.07.017 23928300

[B3] Cheng F. Y. Yang Y. R. Chen L. M. Wu Y. R. Cheng S. J. Wang R. Y. (2016). Positive effects of specific exercise and novel turning-based treadmill training on turning performance in individuals with Parkinson’s disease: A randomized controlled trial. *Sci. Rep.* 6:33242. 10.1038/srep33242 27628128 PMC5023848

[B4] Cubo E. Bernard B. Leurgans S. Raman R. (2000). Cognitive and motor function in patients with Parkinson’s disease with and without depression. *Clin. Neuropharmacol.* 23 331–334. 10.1097/00002826-200011000-00006 11575867

[B5] Curran P. J. Obeidat K. Losardo D. (2010). Twelve frequently asked questions about growth curve modeling. *J. Cogn. Dev.* 11 121–136. 10.1080/15248371003699969 21743795 PMC3131138

[B6] Darweesh S. K. Verlinden V. J. Stricker B. H. Hofman A. Koudstaal P. J. Ikram M. A. (2017). Trajectories of prediagnostic functioning in Parkinson’s disease. *Brain* 140 429–441. 10.1093/brain/aww291 28082300

[B7] de Craen A. J. Heeren T. J. Gussekloo J. (2003). Accuracy of the 15-item geriatric depression scale (GDS-15) in a community sample of the oldest old. *Int. J Geriatr. Psychiatry* 18 63–66. 10.1002/gps.773 12497557

[B8] de la Riva P. Smith K. Xie S. X. Weintraub D. (2014). Course of psychiatric symptoms and global cognition in early Parkinson disease. *Neurology* 83 1096–1103. 10.1212/WNL.0000000000000801 25128183 PMC4166362

[B9] Dubovsky S. L. Ghosh B. M. Serotte J. C. Cranwell V. (2021). Psychotic depression: Diagnosis, differential diagnosis, and treatment. *Psychother. Psychosom.* 90 160–177. 10.1159/000511348 33166960

[B10] Fang H. Tu S. Sheng J. Shao A. (2019). Depression in sleep disturbance: A review on a bidirectional relationship, mechanisms and treatment. *J. Cell Mol. Med.* 23 2324–2332. 10.1111/jcmm.14170 30734486 PMC6433686

[B11] He Z. Lu F. Sheng W. Han S. Long Z. Chen Y. (2019). Functional dysconnectivity within the emotion-regulating system is associated with affective symptoms in major depressive disorder: A resting-state fMRI study. *Aust. N. Z. J. Psychiatry* 53 528–539. 10.1177/0004867419832106 30813750

[B12] Heinzel S. Berg D. Gasser T. Chen H. Yao C. Postuma R. B. (2019). MDS task force on the definition of Parkinson’s disease. Update of the MDS research criteria for prodromal Parkinson’s disease. *Mov. Disord.* 34 1464–1470. 10.1002/mds.27802 31412427

[B13] Hongxia J. J. (2023). Expert consensus on full-cycle rehabilitation of dysfunction in elderly Parkinson’s disease. *Chin. Med. J.* 58 134–140. 10.3969/j.issn.1008-1070.2023.02.006

[B14] Hu L. T. Bentler P. M. (1999). Cutoff criteria for fit indexes in covariance structure analysis: Conventional criteria versus new alternatives. *Struct. Equ. Model.* 6 1–55. 10.1080/10705519909540118

[B15] Iranzo A. Gelpi E. Tolosa E. Molinuevo J. L. Serradell M. Gaig C. (2014). Neuropathology of prodromal Lewy body disease. *Mov. Disord.* 29 410–415. 10.1002/mds.25825 24488760

[B16] Jellinger K. A. (1999). Post mortem studies in Parkinson’s disease–is it possible to detect brain areas for specific symptoms? *J. Neural Transm. Suppl.* 56 1–29. 10.1007/978-3-7091-6360-3_1 10370901

[B17] Kashif M. Ahmad A. Bandpei M. A. M. Gilani S. A. Hanif A. Iram H. (2022). Combined effects of virtual reality techniques and motor imagery on balance, motor function and activities of daily living in patients with Parkinson’s disease: A randomized controlled trial. *BMC Geriatr.* 22:381. 10.1186/s12877-022-03035-1 35488213 PMC9055773

[B18] LaHue S. C. Comella C. L. Tanner C. M. (2016). The best medicine? The influence of physical activity and inactivity on Parkinson’s disease. *Mov. Disord.* 31 1444–1454. 10.1002/mds.26728 27477046 PMC9491025

[B19] Lees A. J. Hardy J. Revesz T. (2009). Parkinson’s disease. *Lancet* 373 2055–2066. 10.1016/S0140-6736(09)60492-X 19524782

[B20] LeWitt P. A. Chaudhuri K. R. (2020). Unmet needs in Parkinson disease: Motor and non-motor. *Parkinsonism Relat. Disord.* 80 S7–S12. 10.1016/j.parkreldis.2020.09.024 33349582

[B21] Liu W. Jin Q. Hao X. M. (2021). Effect of aerobic exercise intervention on the activation degree of HPA axis in depressive rats and its upstream regulation mechanism. *J. Phys. Educ.* 28 138–144. 10.16237/j.cnki.cn44-1404/g8.2021.02.022

[B22] Ma C. L. Su L. Xie J. J. Long J. X. Wu P. Gu L. (2014). The prevalence and incidence of Parkinson’s disease in China: A systematic review and meta-analysis. *J. Neural Transm.* 121 123–134. 10.1007/s00702-013-1092-z 24057652

[B23] Moustafa A. A. Chakravarthy S. Phillips J. R. Crouse J. J. Gupta A. Frank M. J. (2016). Interrelations between cognitive dysfunction and motor symptoms of Parkinson’s disease: Behavioral and neural studies. *Rev. Neurosci.* 27 535–548. 10.1515/revneuro-2015-0070 26982614

[B24] Mulders P. C. R. van Eijndhoven P. F. P. van Oort J. Oldehinkel M. Duyser F. A. Kist J. D. (2022). Striatal connectopic maps link to functional domains across psychiatric disorders. *Transl. Psychiatry* 12:513. 10.1038/s41398-022-02273-6 36513630 PMC9747785

[B25] Munhoz R. P. Moro A. Silveira-Moriyama L. Teive H. A. (2015). Non-motor signs in Parkinson’s disease: A review. *Arq. Neuro Psiquiatria* 73 454–462. 10.1590/0004-282X20150029 26017214

[B26] Nabizadeh F. Pirahesh K. Khalili E. (2022). Olfactory dysfunction is associated with motor function only in tremor-dominant Parkinson’s disease. *Neurol. Sci.* 43 4193–4201. 10.1007/s10072-022-05952-w 35166976

[B27] Poonja S. Miyasaki J. Fu X. Camicioli R. Sang T. Yuan Y. (2021). The trajectory of motor deterioration to death in Parkinson’s disease. *Front. Neurol.* 12:670567. 10.3389/fneur.2021.670567 34484095 PMC8416311

[B28] Postuma R. B. Berg D. Stern M. Poewe W. Olanow C. W. Oertel W. (2015a). MDS clinical diagnostic criteria for Parkinson’s disease. *Mov. Disord.* 30 1591–1601. 10.1002/mds.26424 26474316

[B29] Postuma R. B. Gagnon J. F. Bertrand J. A. Génier Marchand D. Montplaisir J. Y. (2015b). Parkinson risk in idiopathic REM sleep behavior disorder: Preparing for neuroprotective trials. *Neurology* 84 1104–1113. 10.1212/WNL.0000000000001364 25681454 PMC4371408

[B30] Qin Y. Meng D. T. Jin Z. H. Du W. J. Fang B. Y. (2024). Association between autonomic dysfunction with motor and non-motor symptoms in patients with Parkinson’s disease. *J. Neural Transm Gen.* 131 323–334. 10.1007/s00702-024-02745-7 38253927

[B31] Seidel K. Mahlke J. Siswanto S. Krüger R. Heinsen H. Auburger G. (2015). The brainstem pathologies of Parkinson’s disease and dementia with Lewy bodies. *Brain Pathol.* 25 121–135. 10.1111/bpa.12168 24995389 PMC4397912

[B32] Titova N. Chaudhuri K. R. (2018). Non-motor Parkinson disease: New concepts and personalised management. *Med. J. Aust.* 208 404–409. 10.5694/mja17.00993 29764353

[B33] Walter U. Heilmann R. Kaulitz L. Just T. Krause B. J. Benecke R. (2015). Prediction of Parkinson’s disease subsequent to severe depression: A ten-year follow-up study. *J. Neural Transm.* 122 789–797. 10.1007/s00702-014-1313-0 25217967

[B34] Weintraub D. Aarsland D. Chaudhuri K. R. Dobkin R. D. Leentjens A. F. Rodriguez-Violante M. (2022). The neuropsychiatry of Parkinson’s disease: Advances and challenges. *Lancet Neurol.* 21 89–102. 10.1016/S1474-4422(21)00330-6 34942142 PMC8800169

[B35] Weintraub D. Mamikonyan E. (2019). The neuropsychiatry of Parkinson disease: A perfect storm. *Am. J. Geriatr. Psychiatry* 27 998–1018. 10.1016/j.jagp.2019.03.002 31006550 PMC7015280

[B36] Xu J. Wei Q. Bai T. (2020). Electroconvulsive therapy modulates functional interactions between submodules of the emotion regulation network in major depressive disorder. *Transl. Psychiatry* 10:271. 10.1038/s41398-020-00961-9 32759936 PMC7406501

[B37] Xu X. Fu Z. Le W. (2019). Exercise and Parkinson’s disease. *Int. Rev. Neurobiol.* 147 45–74. 10.1016/bs.irn.2019.06.003 31607362

[B38] Zahodne L. B. Marsiske M. Okun M. S. Rodriguez R. L. Malaty I. Bowers D. (2012). Mood and motor trajectories in Parkinson’s disease: Multivariate latent growth curve modeling. *Neuropsychology* 26 71–80. 10.1037/a0025119 22142359 PMC3296901

[B39] Zesiewicz T. A. (2019). Parkinson disease. *Continuum* 25 896–918. 10.1212/CON.0000000000000764 31356286

