# Supplementary material for: Motor dysfunction in Parkinson’s patients: depression differences in a latent growth model
Source: Front Aging Neurosci. 2024 Jun 3;16:1393887. doi: 10.3389/fnagi.2024.1393887 (PMC11181910; doi:10.3389/fnagi.2024.1393887)
Supplement: Supplementary file 1 [file Data_Sheet_1.docx]

Code-motor

**Linear latent growth model：**

DATA:

FILE IS C:\Users\Bepanda\Desktop\finmotor.dat;

VARIABLE:

MISSING ARE ALL (-99);

NAMES ARE PATNO age SEX educ race fampd ageonset duration updrs3BL

updrs3V04 updrs3V06 updrs3V08 updrs3V10 gdsBL gdsV04

gdsV06 gdsV08 gdsV10;

usevar=updrs3BL updrs3V04 updrs3V06 updrs3V08 updrs3V10;

analysis:estimator=mlr; !MLR is Maximum Likelihood RobustEstimator

model:interc slope | updrs3BL@0 updrs3V04@1 updrs3V06@2 updrs3V08@3 updrs3V10@4 ;

output:sampstat stdyx patterns; !patterns Is the missing pattern of missing values.

**Quadratic latent growth model：**

DATA:

FILE IS C:\Users\Bepanda\Desktop\finmotor.dat;

VARIABLE:

MISSING ARE ALL (-99);

NAMES ARE PATNO age SEX educ race fampd ageonset duration updrs3BL

updrs3V04 updrs3V06 updrs3V08 updrs3V10 gdsBL gdsV04

gdsV06 gdsV08 gdsV10;

usevar=updrs3BL updrs3V04 updrs3V06 updrs3V08 updrs3V10;

analysis:estimator=mlr; !MLR is Maximum Likelihood RobustEstimator

MODEL:

F0 by updrs3BL@1 updrs3V04@1 updrs3V06@1 updrs3V08@1 updrs3V10@1 ;!The intercept growth factor is defined, and the fixed factor load is all 1

F1 by updrs3BL@0 updrs3V04@1 updrs3V06@2 updrs3V08@3 updrs3V10@4 ;!The linear slope growth factor was defined with a fixed factor load of 012345

F2 by updrs3BL@0 updrs3V04@1 updrs3V06@4 updrs3V08@9 updrs3V10@16 ; !The second order slope growth factor was defined and the fixed factor load was 0 1 4 9 16 25

[updrs3BL-updrs3V10@0];

[F0 F1 F2];!The Mplus is required to output the mean value of the potential growth factor

OUTPUT:TECH4；

**Quadratic latent growth model（Adding the time-varying covariates）：**

DATA:

FILE IS C:\Users\Bepanda\Desktop\finmoto1.dat;

VARIABLE:

MISSING ARE ALL (-99);

NAMES ARE PATNO updrs3BL updrs3V04 updrs3V06 updrs3V08 updrs3V10

gdsBL gdsV04 gdsV06 gdsV08 gdsV10;

usevar=gdsBL gdsV04 gdsV06 gdsV08 gdsV10

updrs3BL updrs3V04 updrs3V06 updrs3V08 updrs3V10;

analysis:estimator=mlr; MLR is Maximum Likelihood RobustEstimator

MODEL:

F0 by updrs3BL@1 updrs3V04@1 updrs3V06@1 updrs3V08@1 updrs3V10@1 ; !The intercept growth factor is defined, and the fixed factor load is all 1

F1 by updrs3BL@0 updrs3V04@1 updrs3V06@2 updrs3V08@3 updrs3V10@4 ; !The linear slope growth factor was defined with a fixed factor load of 012345

F2 by updrs3BL@0 updrs3V04@1 updrs3V06@4 updrs3V08@9 updrs3V10@16 ; !The second order slope growth factor was defined and the fixed factor load was 0 1 4 9 16 25

updrs3BL on gdsBL;

updrs3V04 on gdsV04;

updrs3V06 on gdsV06;

updrs3V08 on gdsV08 ;

updrs3V10 on gdsV10;

[updrs3BL-updrs3V10@0];

[F0 F1 F2];!The Mplus is required to output the mean value of the potential growth factor

OUTPUT:TECH4;

**Parallel LGM of depression and motor dysfunction**

DATA:

FILE IS C:\Users\Bepanda\Desktop\finmoto1.dat;

VARIABLE:

MISSING ARE ALL (-99);

NAMES ARE PATNO updrs3BL updrs3V04 updrs3V06 updrs3V08 updrs3V10

gdsBL gdsV04 gdsV06 gdsV08 gdsV10;

usevar=gdsBL gdsV04 gdsV06 gdsV08 gdsV10

updrs3BL updrs3V04 updrs3V06 updrs3V08 updrs3V10;

analysis:estimator=mlr; !MLR is Maximum Likelihood RobustEstimator

MODEL:

!Build latent variables

Igds BY gdsBL@1 gdsV04@1 gdsV06@1 gdsV08@1 gdsV10@1;

Sgds BY gdsBL@0 gdsV04@1 gdsV06@2 gdsV08@3 gdsV10@4 ;

Qgds BY gdsBL@0 gdsV04@1 gdsV06@4 gdsV08@9 gdsV10@16 ;

Iupdrs BY updrs3BL@1 updrs3V04@1 updrs3V06@1 updrs3V08@1 updrs3V10@1 ;

Supdrs BY updrs3BL@0 updrs3V04@1 updrs3V06@2 updrs3V08@3 updrs3V10@4 ;

Qupdrs BY updrs3BL@0 updrs3V04@1 updrs3V06@4 updrs3V08@9 updrs3V10@16 ;

!Build the path

Igds with Sgds Qgds;

Sgds with Qgds;

Iupdrs with Supdrs Qupdrs;

Supdrs with Qupdrs;

Iupdrs on Igds;

Supdrs on Sgds;

Qupdrs on Qgds;

output:sampstat stdyx ;
